# Dynamic changes of immunocyte subpopulations in thermogenic activation of adipose tissues

**DOI:** 10.3389/fimmu.2024.1375138

**Published:** 2024-05-15

**Authors:** Yuqing Ye, Huiying Wang, Wei Chen, Zhinan Chen, Dan Wu, Feng Zhang, Fang Hu

**Affiliations:** National Clinical Research Center for Metabolic Diseases, Key Laboratory of Diabetes Immunology, Ministry of Education, Department of Metabolism and Endocrinology, The Second Xiangya Hospital of Central South University, Changsha, Hunan, China

**Keywords:** immune cells, cold exposure, thermogenesis, adipose tissues, bioinformatics

## Abstract

**Objectives:**

The effects of cold exposure on whole-body metabolism in humans have gained increasing attention. Brown or beige adipose tissues are crucial in cold-induced thermogenesis to dissipate energy and thus have the potential to combat metabolic disorders. Despite the immune regulation of thermogenic adipose tissues, the overall changes in vital immune cells during distinct cold periods remain elusive. This study aimed to discuss the overall changes in immune cells under different cold exposure periods and to screen several potential immune cell subpopulations on thermogenic regulation.

**Methods:**

Cibersort and mMCP-counter algorithms were employed to analyze immune infiltration in two (brown and beige) thermogenic adipose tissues under distinct cold periods. Changes in some crucial immune cell populations were validated by reanalyzing the single-cell sequencing dataset (GSE207706). Flow cytometry, immunofluorescence, and quantitative real-time PCR assays were performed to detect the proportion or expression changes in mouse immune cells of thermogenic adipose tissues under cold challenge.

**Results:**

The proportion of monocytes, naïve, and memory T cells increased, while the proportion of NK cells decreased under cold exposure in brown adipose tissues.

**Conclusion:**

Our study revealed dynamic changes in immune cell profiles in thermogenic adipose tissues and identified several novel immune cell subpopulations, which may contribute to thermogenic activation of adipose tissues under cold exposure.

## 1 Introduction

Cold exposure can affect whole-body energy expenditure in humans (1). Voluntary skeleton muscle contractions are associated with shivering thermogenesis (ST) whereas thermogenic adipose tissues are mainly associated with non-shivering thermogenesis (NST) (2). Studies have reported that activation of NST is beneficial for metabolic health such as reducing adiposity, improving insulin sensitivity, and even tumor suppression (3–6). Adipose tissues, particularly brown adipose tissues (BAT), play essential roles in NST.

Adipose tissues, which are present in various depots throughout the body, are highly dynamic organs (7, 8). BAT, characterized by small multi-chamber lipid droplets and abundant mitochondria, is crucial in NST and energy dissipation (9); whereas white adipose tissue (WAT) is mainly responsible for energy storage. In response to cold exposure or beta-adrenergic receptor activation, beige adipocytes, derived from progenitor cells or trans-differentiation from white adipocytes and mainly located in subcutaneous adipose tissue (SAT), can also dissipate energy and promote thermogenesis (10, 11).

As reported, activation of thermogenic adipose tissues (BAT or beiging of SAT) under cold conditions is an effective method to dissipate energy for heat production, thereby reducing energy storage and the risk of obesity and associated metabolic disorders (12).

Adipose tissues primarily consist of adipocytes and stromal vascular fraction (SVF) cells, including immune cells, endothelial cells, mesenchymal stem cells, and neuronal cells, all of which dynamically interact to maintain the balance of the adipose microenvironment (13). Upon cold exposure, thermogenic brown/beige adipocytes can be regulated by alterations in the immune microenvironment, which is composed of multiple immune cells and stromal cells (14, 15). During the cold adaptation process, type 2 innate lymphoid cells (ILC2s) produce methionine-enkephalin peptide (MetEnk) to induce UCP1 expression, therefore promoting beige adipogenesis (16). Besides, cold stimulation initiates the generation of M2 macrophages, which can synthesize and release Slit3 to promote sympathetic neuron growth and tyrosine hydroxylase (TH) activity, thus leading to the thermogenesis of adipocytes (17). Despite the established importance of immune cells in thermogenic modulation, there are currently no reports of systemic and dynamic changes in immune cells during cold exposure in BAT and SAT.

In this study, using integrative bioinformatics tools and Cibersort and mMCP-counter algorithm, we analyzed the immune infiltration of adipose tissues to identify the dynamic changes in immune cells in BAT and SAT under cold stimulation. Single-cell sequencing (ScRNA-seq) datasets, quantitative real-time PCR (RT-qPCR), flow cytometry and immunofluorescence were employed for validation. Bioinformatics analyses showed comprehensive dynamic changes in immune cell profiles in BAT and SAT, consistent with physical thermogenic activation of adipose tissues under cold stimulation. The experimental results further confirmed that, in BAT, the proportion of naïve and memory T cells and monocytes increased, while the proportion of NK cells decreased compared to that under thermoneutral conditions. Overall, our study provided novel immune cell profiles for energy expenditure in thermogenic adipose tissues.

## 2 Material and methods

### 2.1 Data collection and preprocessing

Different gene expression profiles were obtained from the GEO database of the National Center for Biotechnology Information (NCBI; https://www.ncbi.nlm.nih.gov/geo/). All RNA sequencing data were listed as below: Brown and white adipose tissues: GSE44138 (cold 1d), GSE118849 (cold 3d), GSE148361(cold 4d); Brown adipose tissues: GSE100924 (cold 6h), GSE133050 (cold 6h), GSE164936 (cold 6h), GSE149124 (cold 6h), GSE119964 (cold 6h), GSE119452 (cold 6h/1d/2d), GSE207705 (cold 6h/1d/2d/3d/4d/5d), GSE181123 (cold 6h), GSE147392 (cold 6h), GSE110055 (cold 6h), GSE135391(cold 1d), GSE70734 (cold 3d), GSE148361 (cold 4d), GSE86338 (cold 7d), GSE144186 (cold 7d), GSE178720 (cold 10d), GSE51080 (cold 10d); White adipose tissues: GSE140259 (cold 2d), GSE179385 (cold 3d), GSE148361(cold 4d), GSE164219 (cold 7d), GSE145498 (cold 7d), GSE13432 (cold 7d/35d), GSE110420 (cold 10d), GSE51080 (cold 10d), GSE183000 (cold 14d).

### 2.2 Immune infiltration analysis

Two methods were used for evaluating the infiltration of immune cells in expression profiles. One was the Microenvironment Cell Population counter (mMCP-counter). Based on highly specific transcriptomic markers, 16 immune and stromal murine cell populations were quantified to evaluate their infiltration for the control and cold stimulations using the R package “mMCP-counter” (18). Another method was CIBERSORT (19), which estimated the relative proportion of each relevant cell type using an input matrix of reference gene expression signatures. The proportion of 25 different immune cells between cold and control groups was compared by two-sided statistical tests, where the significant level was set at p < 0.05. The Corrplot package was used for the correlation analysis of infiltrating immune cells.

### 2.3 Reanalysis of ScRNA-seq data

ScRNA-seq data of GSE207706 was downloaded from the GEO database of the NCBI. The gene expression profiles were paired and normalized based on R software (version 6.0.2). Briefly, sequencing data were analyzed using the “Seurat” package following the provided protocol (20). The “SingleR” package and PanglaoDB database were used for cell type annotation (21, 22). Cells were represented with Distributed Stochastic Neighbor Embedding (TSNE) plots (23).

### 2.4 Animal procedures

Male C57Bl/6J mice (8-10 weeks) were either exposed to 4°C for designed periods or at room temperature (RT, 22°C) with free access to water and food under controlled conditions of light and darkness (12h/12h). All animal experiments were approved by the Animal Care and Use Committee of Central South University. Adipose tissue samples were collected for RT-qPCR, flow cytometry or immunofluorescence. The *in vivo* experiments were repeated two times independently.

### 2.5 RT-qPCR

SVFs of BAT and SAT were isolated from mice either at RT or exposed to 4°C (Cold) (1d/3d/7d) as previously reported (24). Briefly, BAT and SAT were collected, sliced into pieces with scissors, and digested in Collagenase II buffer for 30 min at 37°C. Then the suspension was filtered and centrifuged for separation of SVFs from adipocyte fractions. The SVFs were incubated with red cell lysing reagent for 5 min, centrifuged at 500 g for 10 min at 4°C, and washed with PBS. Then total RNA was extracted using the Magzol reagent (Magen, #R4801-01) and synthesized into complementary DNA using the Reverse transcription kit (Accurate Biology, #AG11728). Real-time PCR was performed with gene-specific primers and mixture (Yeason, #11202ES03) on Applied Biosystems™ 7900HT Fast Real-Time PCR. Primers and gene information are shown in **Table S1**.

### 2.6 Flow cytometry

SVF was isolated from male mice as mentioned above. Single-cell suspension was divided into two parts. One part was stained with zombie-APC-Cy7 dye (1:200, Biolegend, #423105) for 10 min and then washed with cell staining buffer (BD Pharmingen#554657). After incubation with FcX blocking (1:100, Biolegend, #156604) for 10 min, cells were stained with specific antibodies. For the analysis of monocytes, suspended cells were stained with CD45-PE (1:100, Biolegend, #147711), Ly6C-AF488 (1:100, Biolegend, #128021), Ly6G-BV510(1:100, Biolegend, #127633) and CD11B-Pacific blue (1:100, Biolegend, #101223). For the analysis of M1 and M2 macrophages, suspended cells were stained with CD45-PerCP-Cyanine5.5 (1:100, Biolegend, #103131), CD11B-Pacific blue (1:100, Biolegend, #101223), F4/80-PE/Cy7 (1:100, Biolegend, #123114), CD206-PE (1:100, Biolegend, #141705), and CD11C-Percp (1:100, Biolegend, #117325). For the analysis of NK and NKT cells, suspended cells were stained with CD45-PE (1:100, Biolegend, #147711), CD3-APC (1:100, Biolegend, #100235), and NK1.1-PE/Cy7 (1:100, Biolegend, #156513). For the analysis of endothelial cells, suspended cells were stained with CD45-PerCP-Cyanine5.5 (1:100, Biolegend, #103131) and CD31-APC (1:100, Biolegend, #102409). The other suspension was incubated with FcX blocking (1:100, Biolegend, #156604) for 10 min, and then the cells were stained with specific antibodies. For the analysis of CD4 and CD8 subpopulations, suspended cells were stained with CD45-PerCP-Cyanine5.5 (1:100, Biolegend, #103131), CD3-FITC (1:100, Biolegend, #100203), CD4-Pacific blue (1:100, Biolegend, #100427), CD8-AF700 (1:100, Biolegend, #100729), CD44-APC (1:100, Biolegend, #103011), and CD62L-APC-Cy7 (1:100, Biolegend, #104427). After washes with a cell staining buffer, the cells were finally incubated with Propidium Iodide Solution-PE (1:100, Biolegend, #421301) before fluorescence-activated cell sorting. Stained cells were sorted by flow cytometer (Cytek Northern Lights 3000 and Agilent Novocyte Advanteon) and data were analyzed with FlowJo software version 10.9.0.

### 2.7 Immunofluorescence assay

Fresh adipose tissues were obtained, fixed, and embedded in paraffin. After antigen repair and hydrogen peroxide blocking, the slides were blocked with a solution containing 3% BSA before incubation with primary antibodies at 4°C overnight. The slides were then treated with horseradish peroxidase (HRP)-conjugated anti-rabbit (Servicebio, GB23303, 1:500) or Alexa Fluor 488-conjugated anti-rabbit (Servicebio, GB25303, 1:400) secondary antibodies for 50 min at room temperature. The primary antibodies included CD44 (Servicebio, GB112054, 1:3000), CD62L (Bioss, bs-1036R, 1:1000), CD31 (Servicebio, GB113151, 1:1000), TH (Servicebio, GB11181, 1:1000), CD11B (Service bio, GB11058, 1:3000), CD115 (Servicebio, GB11581, 1:1000), and NK1.1 (Abcam, AB289542, 1:100). All the fluorescence pictures were captured by a fluorescence microscope (Nikon Eclipse C1) or laser scanning confocal microscope (Zeiss LSM 780).

### 2.8 Statistical Analysis

Statistical analysis was performed using GraphPad Prism 10.1.1. The results were expressed as mean ± SEM. The p < 0.05 obtained from two-sided tests implied statistically significant differences.

## 3 Results

### 3.1 Data processing

**Figure 1:**
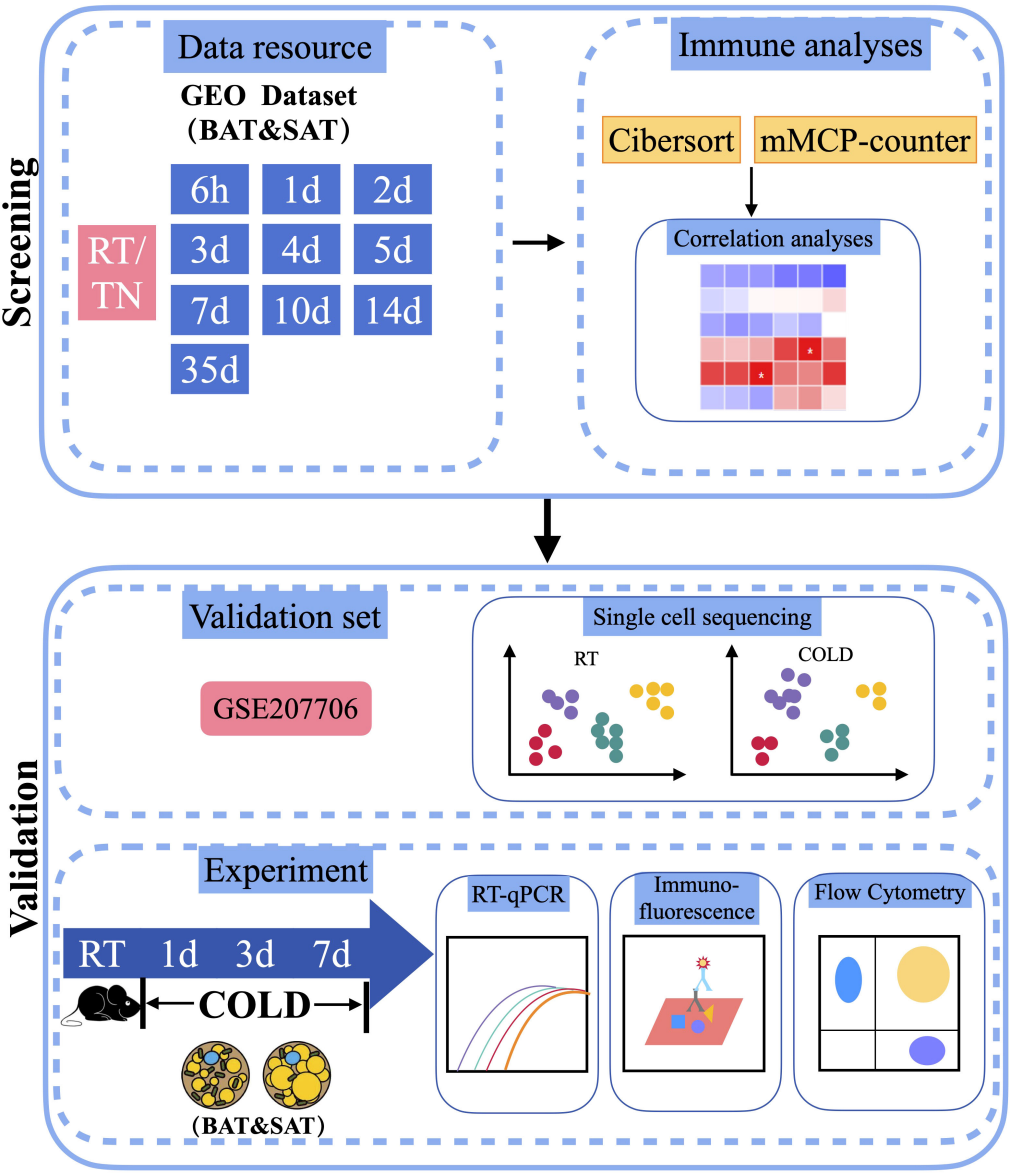
Schematic plot of the workflow of the analysis. RT, Room Temperature; TN, Thermal Neutrality.

To determine changes in different immune cells in the BAT or SAT after different periods of cold exposure, we downloaded distinct datasets of BAT and SAT from the GEO database of the NCBI. The dataset analysis strategy is shown in **Figure 1**. After batch normalization, all datasets were analyzed with two immune algorithms. Significantly different immune cells (and several stromal cells) are listed in **Table 1**.

**Table 1:** Changes in immune cells analyzed by Cibersort and mMCP-Counter algorithms.

BAT	Cibersort	mMCP	Data type	Mice strains	Age	Gender
4-6h(RT-COLD)						
GSE100924	/	↑:Monocytes ↓:Vessels, Endothelial	array	C57BL/6	10w	/
GSE133050	↑:M0 Mø, CD4 T memory ↓:M1 Mø, M2 Mø	↑:Monocytes ↓:T, B derived, Endothelial	High throughput sequencing	C57BL/6	10-20w	male and female
GSE164936	/	↑:Fibroblasts	High throughput sequencing	C57BL/6	12w	female
GSE149124	↑:CD8 T memory, M0 Mø, CD4 T naïve, Th1, M1 Mø, NK ↓:NK actived, DC actived, Eosinophil, Treg	↓:CD8 T, Mast, Eosinophils, Basophils, Neutrophils	High throughput sequencing	C57BL/6	12w	male
GSE119964	↑:Plasma, M0 Mø, γδT ↓:CD8 T Naïve, NK Resting	↑:Monocytes ↓:Vessels	High throughput sequencing	C57BL/6	8-12w	male
GSE119452	↑:M1 Mø	/	High throughput sequencing	C57BL/6	/	male
GSE207705	↑:CD4 T Naïve, Monocyte ↓:CD8 Tmemory	↑:Monocytes ↓:Vessels, Lymphatics, Fibroblast	High throughput sequencing	C57BL/6	8–14w	male
4-6h(TN-COLD)						
GSE164936#	↑:Mast, DC immature. ↓:Monocyte	↑:Monocytes, Fibroblasts ↓:Lymphatics, Endothelial, T, NK	High throughput sequencing	C57BL/6	12w	female
GSE181123	↑:B memory, Plasma , Th1, M0 Mø, M1 Mø, CD4 T Naïve ↓: CD8 T naïve, Treg, Eosinophil, NK Resting, γδT, DC actived	↑:Monocytes / macrophages ↓:Monocytes, Mast, Eosinophils, Vessels, Lymphatics, Basophils, CD8 T	High throughput sequencing	C57BL/6	10w	male
GSE147392	↑:M2 Mø ↓:Th17	↓:B derived, Monocytes, Fibroblasts	High throughput sequencing	C57BL/6	12–16w	male
GSE110055	↑:M0 Mø, Monocyte ↓:Plasma	↑:Monocytes ↓:Vessels, Endothelial	High throughput sequencing	C57BL/6	11-12w	male
1d(RT-COLD)						
GSE119452**	/	↑:Monocytes	High throughput sequencing	C57BL/6	/	male
GSE207705*	↑:CD4 T Naïve ↓:CD8 T Memory	↑:Monocytes ↓:Fibroblasts	High throughput sequencing	C57BL/6	8–14w	male
GSE135391	↓:B Memory	↑:Monocytes ↓:NK, Vessels	High throughput sequencing	C57BL/6	12w	male
GSE44138	↑:M0 Mø ↓:Plasma	/	array	C57BL/6	/	female
2d(RT-COLD)						
GSE119452**	↑:Monocytes ↓:CD8 T Memory, CD4 T Memory	↑:Monocyte, Vessels	High throughput sequencing	C57BL/6	/	male
GSE207705*	↑:CD4 T Naïve ↓:CD8 T Memory	↑:Monocytes ↓:Lymphatics	High throughput sequencing	C57BL/6	8–14w	male
3d(RT-COLD)						
GSE70437	↑:M0 Mø, NK Actived ↓:NK Resting, B Naïve	↑:Monocytes, Vessels	High throughput sequencing	C57BL/6	/	male
GSE207705*	↑:M1 Mø ↓:NK Resting, CD8 T Memory	↑:Monocytes, Vessels	High throughput sequencing	C57BL/6	8–14w	male
3d(TN-COLD)						
GSE118849	↓:γδT	↑:Monocytes ↓:T, NK	High throughput sequencing	C57BL/6	8-12w	male
4d(RT-COLD)						
GSE148361	↑:NK Resting ↓:γδT	↑:Monocytes, Vessels	array	C57BL/6	8w	male
GSE207705*	↑:CD4 T Naïve ↓:CD8 T Memory	↑:Monocytes	High throughput sequencing	C57BL/6	8–14w	male
5d(RT-COLD)						
GSE207705*	↓:CD8 T Memory	↑:Monocytes ↓:Endothelial, Fibroblasts	High throughput sequencing	C57BL/6	8–14w	male
7d(RT-COLD)						
GSE86338	↑:M0 Mø, DC Immature, DC Actived	↑:Monocytes/macrophages, Monocytes, Fibroblasts ↓:CD8 T, B derived	High throughput sequencing	C57BL/6	12w	male
GSE144186	/	↑:B Memory	High throughput sequencing	C57BL/6	8–12w	male
10d(RT-COLD)						
GSE178720	↑:DC Immature ↓:CD8 T Memory, γδT	↑:B derived, Monocytes, Vessels ↓:Fibroblasts ,T	High throughput sequencing	C57BL/6	8–12w	male
10d(TN-COLD)						
GSE51080	↑:CD8 T Actived ↓:NK Resting	↑:Eosinophils. ↓:Vessels, Endothelial, Monocytes / macrophages, Mast	array	129Sv	10w	female

SAT	Cibersort	mMCP	Data type	Mice strains	Age	Gender
1d(RT-COLD)						
GSE44138##	↓:CD8 T Actived	/	array	C57BL/6	/	female
2d(RT-COLD)						
GSE140259	↑:Mast ↓:Plasma	↑:Monocytes	High throughput sequencing	C57BL/6	9w	male and female
3d(TN-COLD)						
GSE179385	/	↑:Monocytes ↓:T, CD8 T, NK, Eosinophils, Vessels	High throughput sequencing	C57BL/6	10–12w	male
GSE118849###	↑:CD8 T Naïve	↓:Monocytes / macrophages, T, CD8 T, B derived, B Memory, Eosinophils, Vessels, Endothelial, Fibroblasts	High throughput sequencing	C57BL/6	8-12w	male
4d(RT-COLD)						
GSE148361#	↑:CD8 T memory, Treg ↓:M1 Mø	↑:Monocytes ↓:Monocytes / macrophages, Vessels, Endothelial, Fibroblasts	array	C57BL/6	8w	male
7d(RT-COLD)						
GSE164219	↓:γδT	↑:Eosinophils, Fibroblasts ↓:Monocytes	High throughput sequencing	C57BL/6	8w	male
GSE145498	/	↓:Eosinophils	array	C57BL/6	9m	/
7d(TN-COLD)						
GSE13432	↑:Treg ↓:CD4 T Naïve, NK Resting	↑:Eosinophils, Endothelial ↓:Monocytes / macrophages, T, NK	array	C57BL/6	6-8w	male
10d(TN-COLD)						
GSE110420	↑:M1 Mø, Th17, DC Immature	↑:Monocytes ↓:Endothelial, Monocytes/ macrophages	array	C57BL/6	12–16w	male
GSE51080***	/	/	array	129Sv	10w	female
14d(RT-COLD)						
GSE183000	↑:Th17, γδT	↓:Eosinophils	High throughput sequencing	C57BL/6	/	/
35d(TN-COLD)						
GSE13432*	↑:B Naïve, CD4 T Memory, T ↓:Plasma, CD8 T Naïve, M1 Mø, CD4 T Naïve, Monocyte, DC Immature	↑:Eosinophils, Vessels ↓:Monocytes / macrophages, T, CD8 T, NK, B derived, Fibroblasts	array	C57BL/6	6-8w	male

Mø, Macrophages. *, **, ***, #, ##, ### repetition of datasets.

### 3.2 Immune cell infiltration and correlation analysis in BAT and SAT

To screen the potential immune cells in thermogenic adipose tissues of mice under different periods of cold exposure, we adopted two well-recognized immune cell screening algorithms. One was the Cibersort algorithm, which measured the proportion of 25 types of immune cells. The other was the mMCP-counter algorithm, which analyzed the abundance of 16 types of murine immune and stromal cells. Consistent with the thermogenic activation of brown fat in acute cold (25, 26), most datasets showed changes started as early as a cold duration of 6h in BAT. These algorithm analyses revealed that beige adipogenesis might occur during longer periods of cold exposure, as 35d long-term cold acclimation resulted in more significant changes in immune cells in SAT (**Table 1**). Next, we focused on several major types of immune cells or stromal cells and pooled the same cold duration datasets to reanalyze their proportions or abundances over time using Cibersort and mMCP-counter methods.

**Figure 2:**
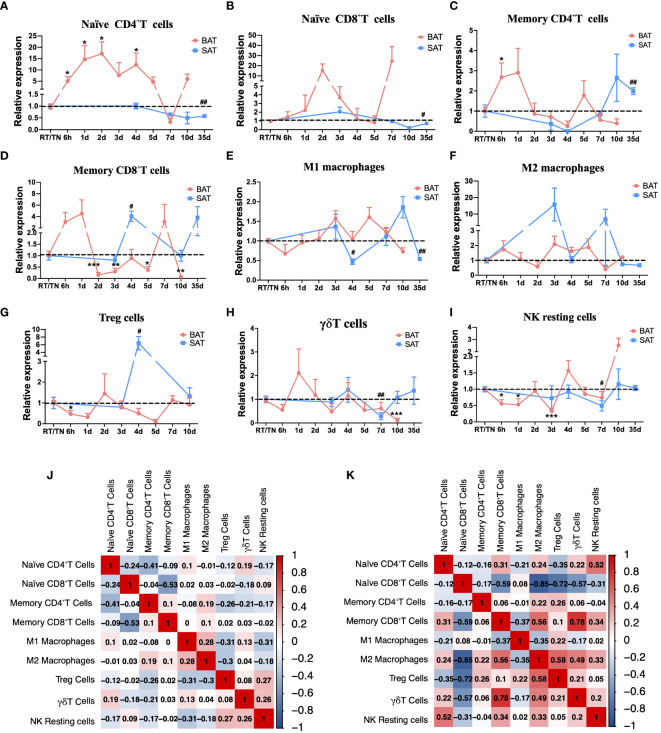
Cibersort analysis of immune cell changes during different cold periods in BAT and SAT. Dynamic expression of naïve CD4^+^T cells **(A)**, naïve CD8^+^T cells **(B)**, memory CD4^+^T cells **(C)**, memory CD8^+^T cells **(D)**, M1 macrophages **(E)**, M2 macrophages **(F)**, Treg cells **(G)**, γδT cells **(H)**, and NK resting cells **(I)** in different cold periods in BAT and SAT. **(J)** Correlation analysis of these immune cells in BAT. **(K)** Correlation analysis of these immune cells in SAT. Statistical data were assessed by unpaired two-tailed Student’s t test. */#P<0.05, **/##P<0.01, ***/###P<0.001, ****/####P<0.0001. BAT, Brown Adipose Tissues; SAT, Subcutaneous Adipose Tissues.

The Cibersort algorithm analysis suggested that in BAT, the proportions of naïve CD4^+^ T cells and naïve CD8^+^ T cells increased throughout most periods of cold exposure compared with that at RT (**Figures 2A, B**). Memory CD4^+^ T cells and memory CD8^+^ T cells displayed similar dynamic change trends (**Figures 2C, D**). In SAT, the proportion of memory CD8^+^T cells, M1 and M2 macrophages, and Treg cells significantly changed in a cold duration of 4d compared to other cold periods (**Figures 2D-G**). Besides, alterations of these immune cells (such as M2 macrophages, Treg cells, and γδT cells) occurred later in SAT than those in BAT during different cold periods, and the cellular dynamics of the two types of adipose tissues were similar during the cold periods (**Figures 2F-H**). Moreover, the proportion of NK resting cells under all cold exposure periods was lower than that at RT in SAT (**Figure 2I**). Due to these changes in immune cells, the corrplot package on R studio was further used to analyze the relationship among these immune cells to explore their possible synergistic or antagonistic interactions. In BAT, these immune cells were less closely related (**Figure 2J**). In SAT, there was an inverse correlation between M2 macrophages and naïve CD8^+^T cells (**Figure 2K**).

**Figure 3:**
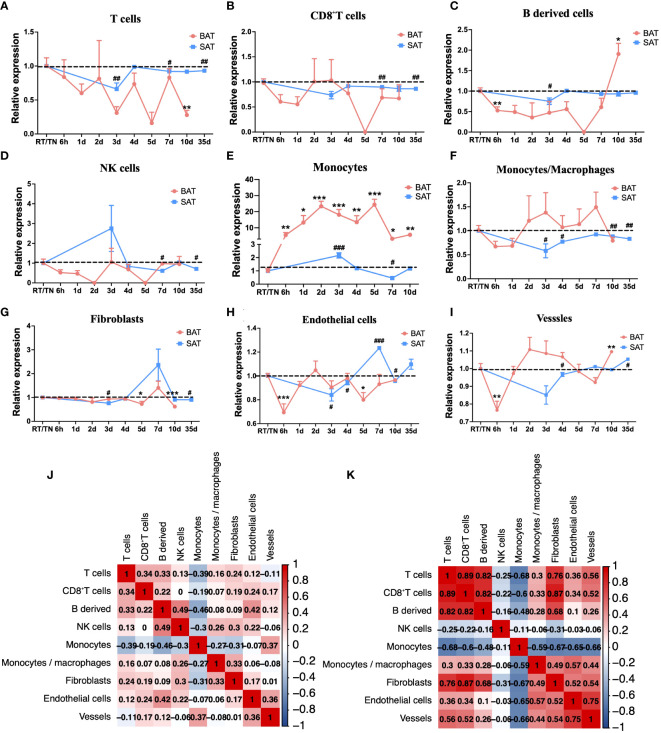
mMCP-counter analysis of immune cell changes during different cold periods in BAT and SAT. Dynamic expression of T cells **(A)**, CD8 T cells **(B)**, B-derived cells **(C)**, NK cells **(D)**, Monocytes **(E)**, Monocytes/macrophages **(F)**, Fibroblasts **(G)**, Endothelial cells **(H)**, and Vessels **(I)** in different cold periods in BAT and SAT. **(J)** Correlation analysis of these immune cells in BAT. **(K)** Correlation analysis of these immune cells in SAT. Statistical data were assessed by unpaired two-tailed Student’s t test. */#P<0.05, **/##P<0.01, ***/###P <0.001, ****/####P<0.0001. BAT, Brown Adipose Tissues; SAT, Subcutaneous Adipose Tissues.

The mMCP-counter algorithm analysis disclosed that the abundance of T cells, CD8^+^T cells, B-derived cells, and NK cells was lower than that at RT or TN in all periods of cold exposure (**Figures 3A-D**), while the abundance of monocytes increased at all cold periods in BAT (**Figure 3E**). Intriguingly, during most cold periods (from 2d to 7d), the changes in monocytes/macrophages in BAT and SAT were opposite (**Figure 3F**), whereas the abundance changes of fibroblasts in these two types of adipose tissues were similar (**Figure 3G**). In addition, endothelial cells and vessels showed similar patterns in both BAT and SAT (**Figures 3H, I**). In SAT, however, except for monocytes and NK cells, the abundance of other immune cells or stromal cells (such as T cells, CD8^+^T cells, B-derived cells, fibroblasts, endothelial cells, and vessels) was significantly decreased at 3 d and then gradually increased in longer periods of cold exposure (**Figures 3A-C, G-I**). Finally, the relationship between immune cells in BAT and SAT was evaluated. In BAT, they were not correlated (**Figure 3J**), but in SAT, CD8^+^T cells and B-derived cells were highly positively correlated, consistent with the abundance changes in BAT and SAT. In contrast, monocytes and T cells were negatively correlated (**Figure 3K**).

### 3.3 ScRNA-seq analysis of major immune cell changes in BAT

**Figure 4:**
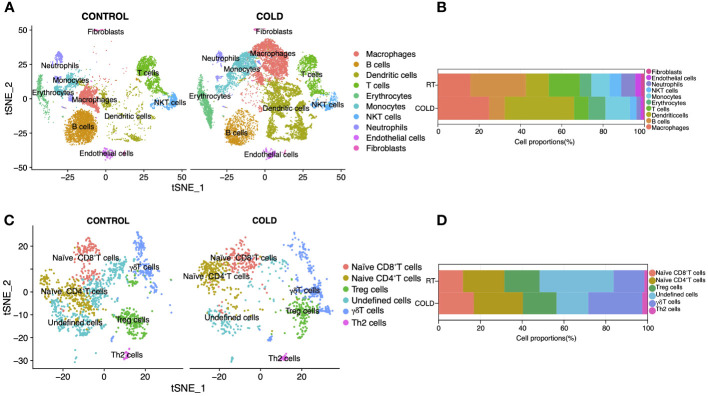
Reanalysis of Single-cell sequencing data (GSE207706) in BAT upon 4d-cold exposure. **(A)** Unsupervised clustering of Lineage^+^ stromal vascular fraction (SVF), shown as t-distributed stochastic neighbor embedding (tSNE). **(B)** The proportion of each cell type in SVF under cold adaptation. **(C)** Unsupervised clustering of T cells shown as t-distributed stochastic neighbor embedding (tSNE). **(D)** The proportion of each cell type in cold adaptation of T cells.

To validate bulk RNA sequencing results, we collected ScRNA-seq datasets (GSE207706) of specific immune cells in BAT upon 4-d cold stimulation. We identified ten clusters within lineage-positive cells (**Figure 4A**) and presented the top 10 marker genes for each cluster in **Supplementary Figure S1A**. Consistent with those mMCP-counter algorithm results, we found that the proportions of T cells and B cells decreased within lineage-positive cells, while the proportion of monocytes increased (**Figure 4B**). Additionally, there was a significant increase in the proportion of other immune cells such as macrophages, dendritic cells, and erythrocytes, while the proportion of NKT cells, neutrophils, endothelial cells and fibroblasts showed a decrease. Subsequently, we further analyzed the T cell cluster within lineage-positive cells and classified it into ten subclusters (**Figure 4C**), presenting their top markers in **Supplementary Figure S1B**; among which naïve CD4^+^T cells increased upon 4d cold exposure, consistent with the above immune cell analysis by Cibersort (**Figure 4D**).

### 3.4 Validation of immune cell changes in adipose tissues under different periods of cold exposure

**Figure 5:**
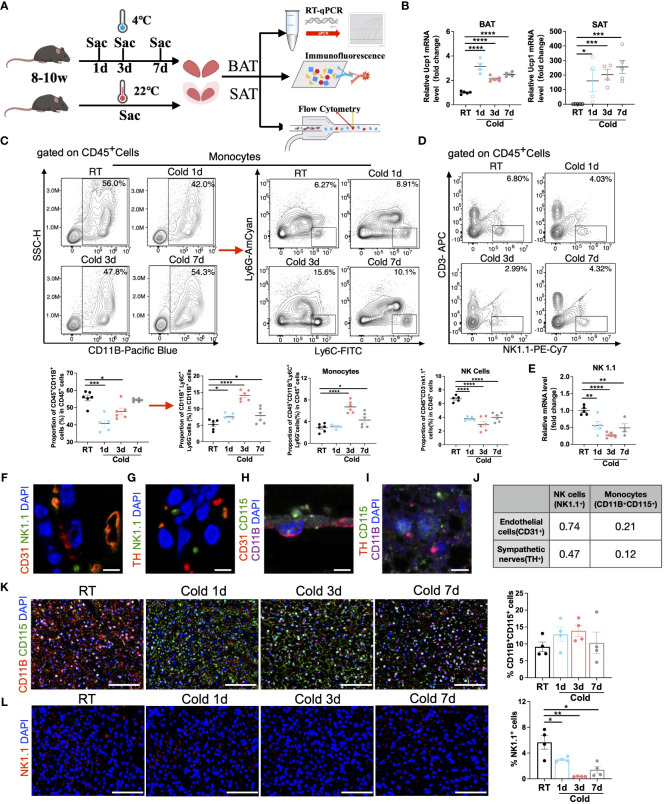
Validation of the changes in monocytes and NK cells under cold exposure. **(A)** Flow chart of the experiment. 8-10w male mice were divided into four groups, and brown and white adipose tissues were collected separately at RT and upon 1d, 3d, and 7d cold exposure for RT-qPCR, immunofluorescence, and flow cytometry. **(B)** UCP1 mRNA expression in BAT (left) and SAT (right) of mice at RT and upon 1d-3d-7d cold exposure. **(C)** Representative flow cytometry plots and quantification of the proportion of monocytes in CD45^+^ cells in BAT of mice at RT and upon 1d-3d-7d cold exposure. **(D)** Representative flow cytometry plots and quantification of the proportion of NK cells in CD45^+^ cells in BAT of mice at RT and upon 1d-3d-7d cold exposure. **(E)** NK1.1 mRNA expression in BAT SVF of mice at RT and upon 1d-3d-7d cold exposure. **(F, G)** Representative images of the localization of NK1.1^+^ NK cells with CD31^+^ endothelial cells **(F)** or TH^+^ sympathetic neurons **(G)** in BAT of mice at RT. Scale bar, 10μm. **(H, I)** Representative images of the localization of CD11B^+^ CD115^+^ monocytes with CD31^+^ endothelial cells **(H)** or TH^+^ sympathetic neurons **(I)** in BAT of mice at RT. Scale bar, 10μm. **(J)** Pearson correlation coefficient of the fluorescence intensity between these cells **(F–I)** in BAT of mice at RT. **(K, L)** Representative images of cold-induced changes in CD11B^+^CD115^+^ monocytes **(K)** and NK1.1^+^ NK cells **(L)** in BAT of mice at RT and upon 1d-3d-7d cold exposure. Scale bar, 50μm. All samples were biologically independent replicates (n=4-6 in each group). Data were presented as mean ± SEM. *P<0.05, **P<0.01, ***P<0.001, ****P<0.0001. RT, Room Temperature; BAT, Brown Adipose Tissues; SAT, Subcutaneous Adipose Tissues; SVF, Stromal Vascular Fraction; Sac, Sacrifice.

To further verify the dynamic changes in immune cells in adipose tissues, we reared the male mice at RT or in cold chambers for 1d, 3d, and 7d, and then collected SVF of BAT and SAT for flow cytometry, immunofluorescence, and RT-qPCR (**Figure 5A**). The expression of Ucp1, a thermogenic marker gene, in BAT and SAT was upregulated in all cold periods, suggesting the effectiveness of cold exposure (**Figure 5B**). Then flow cytometry was performed to analyze the proportion changes of immune cells. As expected in BAT, the proportion of monocytes was increased in cold periods (**Figure 5C** and **Supplementary Figure S2A**), whereas the proportion of NK cells (**Figure 5D** and **Supplementary Figure S2A**) and mRNA expression of NK1.1 (an NK cell marker) (**Figure 5E**) were decreased under all periods of cold stimulation. It is well known that sympathetic neurons play a vital role in the thermogenesis of BAT by releasing norepinephrine, while tyrosine hydroxylase (TH) is an essential marker of sympathetic innervation (27). Vascular endothelial cells provide nutrition and oxygen to brown and beige adipocytes to sustain thermogenesis (28). Given the well-recognized crosstalk of two types of cells with thermogenic adipocytes (28), we further explored the possible localization relationship between these cells and immune cells in BAT. Interestingly, the immunofluorescence intensity of CD31 (an endothelial cell marker) or TH was highly correlated with that of NK1.1 (**Figures 5F, G, J** and **Supplementary Figures S2B, C**). The proximity between these two proteins probably suggested a robust functional interaction between these two types of cells, whereas monocytes (marked by CD11B^+^CD115^+^) were less correlated with either endothelial cells or sympathetic neurons (**Figures 5H-J** and **Supplementary Figures S2D, E**) in BAT. Furthermore, the analysis of immunofluorescence showed an increase in the proportion of monocytes, particularly during 3 days of cold exposure (**Figure 5K** and **Figure S2F**), while the proportion of NK cells decreased across all periods of cold exposure. (**Figure 5L** and **Supplementary Figure S3A**).

Also in SAT, the proportion of monocytes was highly upregulated under cold exposure (**Supplementary Figure S3B**), nevertheless the proportion of NK cells was increased in 1 and 3 days of cold exposure (**Supplementary Figure S3C, D**). Previous studies have shown that iNKT, a subset of NKT cells, can stimulate the production of fibroblast growth factor 21 (FGF21) to regulate the thermogenic browning of white fat (29). Therefore, we also examined changes in NKT cells and observed a decreased proportion in longer periods of cold exposure (3 and 7 days) in SAT (**Supplementary Figures S3C, D**).

**Figure 6:**
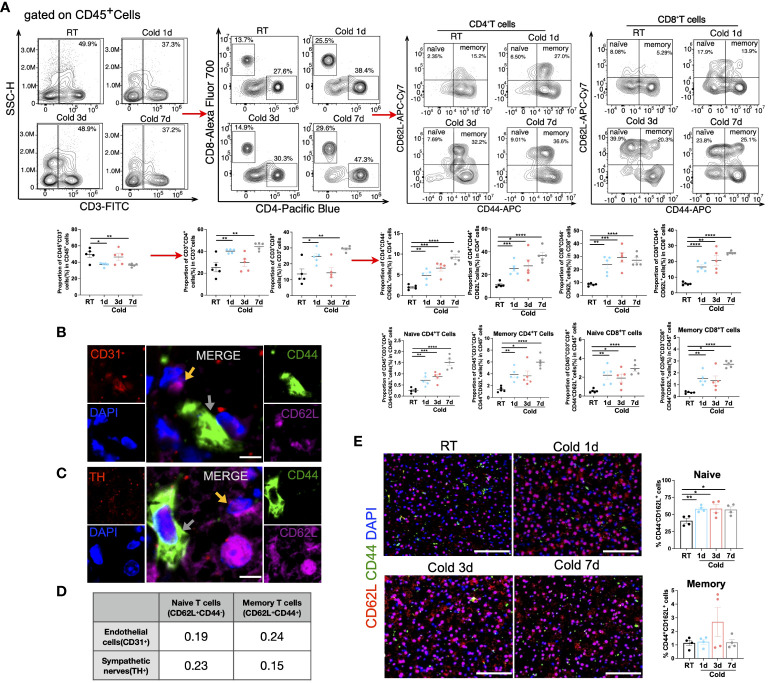
Changes of T cell subpopulations under cold exposure. **(A)** Representative flow cytometry plots and quantification of the proportion of CD4^+^ T cell subpopulations and CD8^+^ T cell subpopulations in CD45^+^ cells in BAT of mice at RT and upon 1d-3d-7d cold exposure. **(B)** Representative images of the localization of CD44^-^CD62L^+^ naïve cells (yellow arrows), CD44^+^CD62L^+^ memory cells (grey arrows), and CD31^+^ endothelial cells in BAT of mice at RT. Scale bar, 10μm. **(C)** Representative images of the localization of CD44^-^CD62L^+^naïve cells (yellow arrows), CD44^+^CD62L^+^ memory cells (grey arrows), and TH^+^ sympathetic neurons in BAT of mice at RT. Scale bar, 10μm. **(D)** Pearson correlation coefficient of the fluorescence intensity between these cells **(B, C)** in BAT of mice at RT. **(E)** Representative images of cold-induced changes in CD44^-^CD62L^+^naïve cells and CD44^+^CD62L^+^ memory cells in BAT of mice at RT and upon 1d-3d-7d cold exposure. Scale bar, 50μm. All samples were biologically independent replicates (n=4-6 in each group). Data were presented as mean ± SEM. *P<0.05, **P<0.01, ***P<0.001, ****P<0.0001. RT, Room Temperature; BAT, Brown Adipose Tissues.

Moreover, flow cytometry manifested that the proportion of naïve T (both CD4^+^ and CD8^+^) cells and memory T (both CD4^+^ and CD8^+^) cells increased in all periods of cold stimulation (**Figure 6A** and **Supplementary Figure S4A**). However, these types of cells were not such close to CD31^+^ endothelial cells or TH^+^ sympathetic neurons (**Figures 6B-D**). The immunofluorescence results implied that the proportion of naïve T cells was increased during 1, 3, or 7d cold exposure, while there was an increased trend in the proportion of memory T cells after 3 days of cold exposure in BAT (**Figure 6E** and **Supplementary Figure S4B**). Additionally, unlike BAT, alterations in naïve and memory T cells were mainly observed after 7 days of cold stimulation in SAT (**Supplementary Figure S4C**).

**Figure 7:**
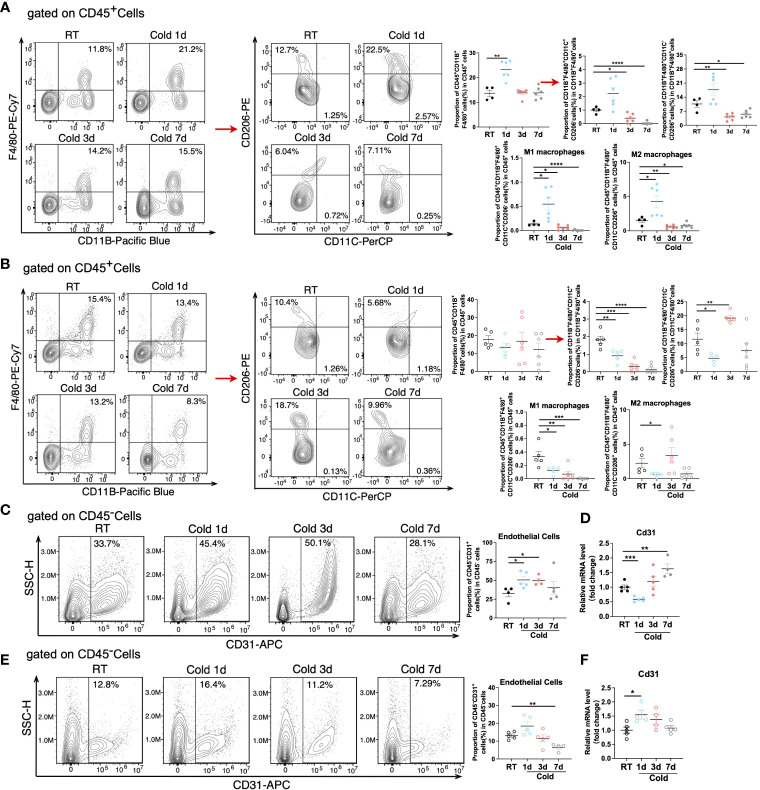
Changes of macrophage subpopulations and endothelial cells under cold exposure. **(A, B)** Representative flow cytometry plots and quantification of the proportion of M1 and M2 macrophages in CD45^+^ cells in BAT **(A)** and SAT **(B)** of mice at RT and upon 1d-3d-7d cold exposure. **(C)** Representative flow cytometry plots and quantification of the proportion of endothelial cells in CD45^-^ cells in BAT of mice at RT and upon 1d-3d-7d cold exposure. **(D)** CD31 mRNA expression in BAT SVF of mice at RT and upon 1d-3d-7d cold exposure. **(E)** Representative flow cytometry plots and quantification of the proportion of endothelial cells in CD45^-^ cells in SAT of mice at RT and upon 1d-3d-7d cold exposure. **(F)** CD31 mRNA expression in SAT SVF of mice at RT and upon 1d-3d-7d cold exposure. All samples were biologically independent replicates (n=4-6 in each group). Data we represented as mean ± SEM. *P<0.05, **P<0.01, ***P<0.001, ****P<0. 0001. RT, Room Temperature; BAT, Brown Adipose Tissues; SAT, Subcutaneous Adipose Tissues; SVF, Stromal Vascular Fraction.

**Figure 8:**
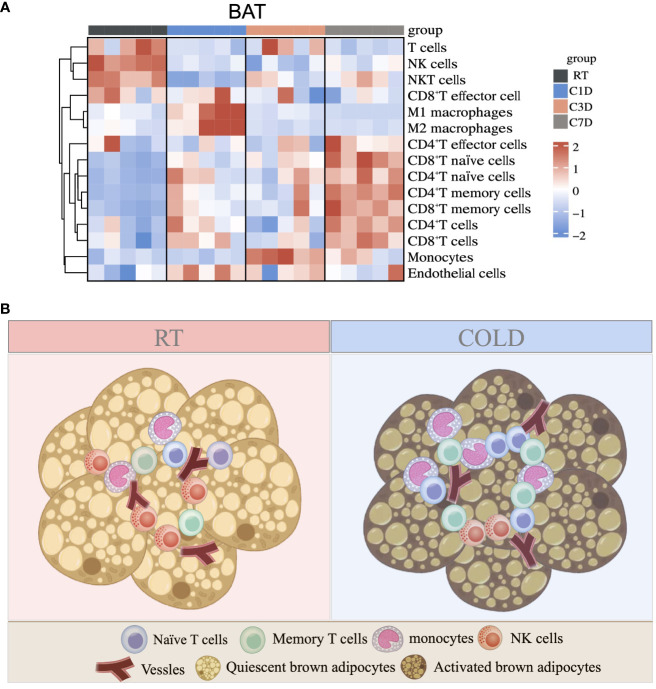
Summary of immune cell changes under cold exposure in BAT. **(A)** Summary of immune cell changes in BAT of mice at RT and upon 1d-3d-7d cold exposure shown as a heatmap. **(B)** Upon cold stimulation, the proportions of monocytes, naïve and memory T cells are increased, while the proportion of NK cells is decreased.

As a predominant subpopulation of immune cells, M1 and M2 macrophages changed dynamically, as their proportion both upregulated in BAT in 1d acute cold and gradually downregulated in 3-7d cold durations (**Figure 7A** and **Supplementary Figure S5A**). In SAT, the proportion of M1 macrophages was reduced in all cold periods, while the proportion of M2 macrophages was enhanced in 3d of cold stimulation (**Figure 7B**). Previous studies have shown the important role of M2 macrophages in thermogenesis. Notably, 6h-cold in BAT and 2d-cold in SAT both promoted the proportion of M2 macrophages (30, 31). Here, our study revealed the dynamic changes of M2 macrophages in different cold durations. Bioinformatics analysis demonstrated that the proportion of endothelial cells, a part of the immune microenvironment, differed from that of other stromal cells or immune cells. Thus, we detected such cells by flow cytometry and RT-qPCR and found that they sustained high levels during the longer period (3-7d) of cold stimulation in BAT (**Figures 7C, D** and **Supplementary Figure S5B**). However, these changes in SAT occurred in the initial 1d cold challenge (**Figures 7E, F**). Besides, ILC2 cells are known for their involvement in thermogenic regulation in SAT under 3 days of cold exposure (32). Their relative markers in both BAT and SAT were further studied. Our results showed that in SAT, the changes in markers IL5, IL13, and IL33 were consistent with those in ILC2 cells under 3-d cold stimulation, but not with those observed in BAT, indicating potential distinct regulations between these two types of adipose tissues (**Supplementary Figures S5C, D**). To summarize, all novel immune cells discovered in BAT and SAT in our studies are shown in **Figure 8** and **Supplementary Figure S5E**.

## 4 Discussion

The activation of BAT or SAT has emerged as a potential strategy for combating obesity and its related metabolic diseases (33, 34). However, the immunomodulation of these adipose tissues, especially under cold exposure, is still not well understood. The absence of thorough investigations into the comprehensive changes in the immune cell profile during cold stimulation is a significant gap that needs to be addressed. In this study, we used two mainstream immune analysis algorithms to comprehensively analyze immune cell datasets in both BAT and SAT from acute (6h) to long-term (35d) cold exposure. The first algorithm was Cibersort, a well-known method with advantages of deep deconvolution and enumeration of cell subsets in tissues with complex compositions; the second was mMCP-counter, which displayed not only the expression of immune cells but also immune-regulated stromal cells. In summary, we have drawn the immune cell profiles in the adipose tissues in response to cold exposure.

As previously reported, M2 macrophages significantly contributed to the browning of SAT (30, 35), while γδT cells promoted BAT innervation and thermogenesis (27). In our study, bioinformatics analysis discovered M2 macrophages and γδT cells in the thermogenic activation of adipose tissues. Besides, our study displayed several important discoveries. First, there were different immune cell patterns in BAT and SAT in response to cold exposure. Most significant changes initially occurred in BAT (especially in 6h or 1d), earlier than those in SAT (especially in 3d). Some significant changes in immune cells, including naïve T (both CD4^+^ and CD8^+^) cells and memory T (both CD4^+^ and CD8^+^) cells were only discovered in BAT, but not in SAT. These differences might reveal inherent unique characteristics of BAT during cold exposure, as brown adipocytes are activated rapidly in response to acute cold challenge, while beige adipocytes in SAT are transdifferentiated from white adipocytes or derived from white pre-adipocytes (26). Immune cells and adipocytes in adipose tissues may interact during these processes, resulting in different changes in immune cells in BAT from those in SAT. In contrast, the changes in certain immune and stromal cells in BAT and SAT were similar, suggesting potential shared regulatory pathways for the interaction between beige and brown adipocytes. These pathways may involve molecular regulations, including adipocyte-specific lineage-determining transcription factors PR domain containing 16 (Prdm16) and peroxisome proliferator-activated receptor γ (PPARγ) coactivator 1 a (PGC1a), which are essential for sustaining the browning process of thermogenic adipocytes. Some non-coding RNAs, such as miRNAs, regulate thermogenesis by binding to the UTR regions of target mRNAs. LncRNAs interact with other important transcription factors such as PGC1a, Early B-cell factor 2 (EBF2), and PPARγ to modulate energy expenditure both in brown and beige adipocytes (13).

Second, our study confirmed that naïve T (both CD4^+^ and CD8^+^) cells and monocytes were the most significantly upregulated immune cells in BAT as predicted by bioinformatics analysis. According to reports, naïve CD4^+^T cells initially induce foxp3^+^ Tregs to facilitate browning in BAT through the Stat6/Pten axis, thus modulating energy expenditure during thermogenesis (36). However, the impact of these naïve CD4^+^T cells on the functions or activities of other cells under long-term cold stimulation remains unclear. Impairment of naïve CD8^+^T cells is associated with dysregulated lipid metabolism in the immune aging process in elderly humans and lipid-altering drug rosiglitazone could restore the responsiveness of naïve CD8^+^ T cells (37). Upon cold stimulation, lipid alteration occurred in BAT regulated the thermogenic process which may imply the possible role of naïve CD8^+^ T cells. Interestingly, further experiments validated that the proportion of memory T cells (both CD4^+^ and CD8^+^) was upregulated during cold periods. Memory CD8^+^T cells exhibit significant mitochondrial spare respiratory capacity (SRC) to produce energy in response to increased stress. This capacity is attributed to their inherent ability for cell-intrinsic lipolysis, which facilitates fatty acid oxidation (FAO) and oxidative phosphorylation in cells (38, 39). These metabolic processes are closely associated with classic thermogenic activation (40, 41). However, the potential involvement of memory CD8^+^T cells in cold-induced thermogenesis has not been documented, which warrants further investigations in future studies. In fact, naïve and memory T cells predominantly rely on FAO rather than glucose-derived pyruvate to produce ATP. Conversely, effector T cells upregulate glycolysis and glutamine oxidation with reduced FAO (42). This supports their possible beneficial roles in thermogenesis.

In our research, scRNA-seq data and experiments confirmed an increased proportion of monocytes in cold exposure. Previous reports have stated that Ccr2^+^ monocytes play a role in recruiting alternatively activated macrophages to induce catecholamine production and control the biogenesis of beige fat under 2d cold exposure in SAT (35), while monocytes in BAT support tissue expansion and matrix remodeling (43). Nonetheless, it is currently unclear whether monocyte recruitment to BAT favors thermogenesis. And in our researches, we observed a rise in the percentage of monocytes during both short-term and prolonged exposure to cold temperatures. This increase may lead to the recruitment of additional cells or the secretion of certain cytokines that could potentially interact with other cells, warranting further examination.

Third, our study implied that the proportion of NK resting cells in cold stimulation was lower than that at RT. NK cells were originally identified as early cytotoxic effectors in anti-tumor and antiviral immune responses. They can secrete diverse proinflammatory cytokines and chemokines to modulate the homeostasis of other immune cells and tissues (44). Prior research has demonstrated that obesity-induced NK cells facilitate stress in adipose tissues, leading to inflammation and insulin resistance by driving the polarization of macrophages toward a proinflammatory phenotype (45, 46). Nonetheless, the involvement of NK cells in adipose tissues during cold exposure remains largely unknown.

There were several limitations in our study. First, although we discovered these types of immune cells in cold exposure, whether and how they function in thermogenic procedures requires further investigation. Second, using certain agonists or neutralizing antibodies to enhance or eliminate these identified immune cells to explore the overall metabolic changes may yield more substantial evidence, which warrants further investigations. Finally, our research was conducted exclusively on mice. Once the necessary resources are available, it is crucial to directly verify these results in human brown and beige adipocytes.

In conclusion, our study analyzed the comprehensive changes in immune cells during different periods of cold exposure in BAT and SAT and discovered several novel subpopulations of immune cells in the thermogenic process. The proportions of monocytes, naïve, and memory T cells were significantly upregulated, while the proportion of NK cells was downregulated in BAT during cold stress. These types of immune cells provide new insights into thermogenic immune modulation in adipose tissues.

## Data availability statement

The datasets presented in this study can be found in online repositories. The names of the repository/repositories and accession number(s) can be found in the article/**Supplementary Material**.

## Ethics statement

The animal study was approved by the University Committee on the Care and Use of Animals of the Central South University, China. The study was conducted in accordance with the local legislation and institutional requirements.

## Author contributions

YY: Writing – original draft, Writing – review & editing. HW: Data curation, Formal Analysis, Writing – review & editing. WC: Investigation, Methodology, Writing – review & editing. ZC: Data curation, Formal Analysis, Software, Writing – review & editing. DW: Resources, Software, Writing – review & editing. FZ: Data curation, Formal Analysis, Writing – original draft. FH: Writing – original draft, Writing – review & editing.
