# Supplementary material for: Dynamic changes of immunocyte subpopulations in thermogenic activation of adipose tissues
Source: Front Immunol. 2024 May 15;15:1375138. doi: 10.3389/fimmu.2024.1375138 (PMC11133676; doi:10.3389/fimmu.2024.1375138)
Supplement: Supplementary file 1 [file DataSheet_1.pdf]

Supplementary Material

Dynamic changes of immunocyte subpopulations in thermogenic activation of adipose tissues

Yuqing Ye, Huiying Wang, Wei Chen, Zhinan Chen, Dan Wu, Feng Zhang, Fang Hu*

*** Correspondence:**
Fang Hu: hu_fang98@csu.edu.cn.

Supplementary Figures and Tables

1 Supplementary Table

Table S1 Primers used for RT-qPCR

	Forward	Reverse
β-Actin	AGCCATGTACGTAGCCATCC	CTCTCAGCTGTGGTGGTGAA
Ucp1	AGGCTTCCAGTACCATTAGGT	CTGAGTGAGGCAAAGCTGATTT
NK1.1	ATGGACACAGCAAGTATCTACCT	AGCTCTCAGGAGTCACTTTATCT
Cd31	ACGCTGGTGCTCTATGCAAG	TCAGTTGCTGCCCATTCATCA

2 Supplementary Figure

Supplementary Figure 1. Marker genes in each cluster of single-cell sequencing.

(A) Top 10 most enriched marker genes in each cluster in Lineage^{+} stromal vascular fraction (SVF) shown as a heatmap. (B) Top 10 most enriched marker genes in each cluster of T cells shown as a heatmap.

Supplementary Figure 2. Validation of the changes in monocytes and NK cells under cold exposure in BAT.

(A) Gating strategy of NK cells and monocytes.

(B) Single channel staining of immunofluorescence in Figure 5F. Scale bar, 10μm.

(C) Single channel staining of immunofluorescence in Figure 5G. Scale bar, 10μm.

(D) Single channel staining of immunofluorescence in Figure 5H. Scale bar, 10μm.

(E) Single channel staining of immunofluorescence in Figure 5I. Scale bar, 10μm.

(F) Single channel staining of immunofluorescence in Figure 5K. Scale bar, 50μm.

Supplementary Figure 3. Validation of the changes in monocytes, NK and NKT cells under cold exposure.

(A) Single channel staining of immunofluorescence in Figure 5L. Scale bar, 50μm.

(B) Representative flow cytometry plots and quantification of the proportion of monocytes in $CD45^+$ cells in SAT of mice at RT and upon 1d-3d-7d cold exposure.

(C) Gating strategy of NKT and NK cells.

(D) Representative flow cytometry plots and quantification of the proportions of NK and NKT cells in $CD45^+$ cells in SAT of mice at RT and upon 1d-3d-7d cold exposure.

All samples were biologically independent replicates (n=4-6 in each group). Data were presented as mean ± SEM. *P<0.05, **P<0.01, ***P<0.001, ****P<0.0001. RT: Room Temperature; SAT: Subcutaneous Adipose Tissues.

Supplementary Figure 4. Validation of the changes in T cell subpopulations under cold exposure.

(A) Gating strategy of T cell subpopulations.

(B) Single channel staining of immunofluorescence in Figure 6E. Scale bar, 50μm.

(C) Representative flow cytometry plots and quantification of the proportions of $CD4^+$T cell subpopulations and $CD8^+$T cell subpopulations in $CD45^+$ cells in SAT of mice at RT and upon 1d-3d-7d cold exposure.

All samples were biologically independent replicates (n=4-6 in each group). Data were presented as mean ± SEM. *P<0.05, **P<0.01, ***P<0.001, ****P<0.0001. RT: Room Temperature; SAT: Subcutaneous Adipose Tissues.

Supplementary Figure 5. Dymanic changes of immune cells under cold exposure.

(A) Gating strategy of macrophage subpopulations.

(B) Gating strategy of endothelial cells.

(C-D) IL-5, IL13 and IL-33 mRNA expression in BAT (C) and SAT (D) of mice at RT and upon 1d-3d-7d cold exposure.

(E) Summary of immune cells changes in SAT of mice at RT and upon 1d-3d-7d cold exposure shown as a heatmap.

All samples were biologically independent replicates (n=4-6 in each group). Data were presented as mean ± SEM. *P<0.05, **P<0.01, ***P<0.001, ****P<0.0001. RT: Room Temperature; SAT: Subcutaneous Adipose Tissues.

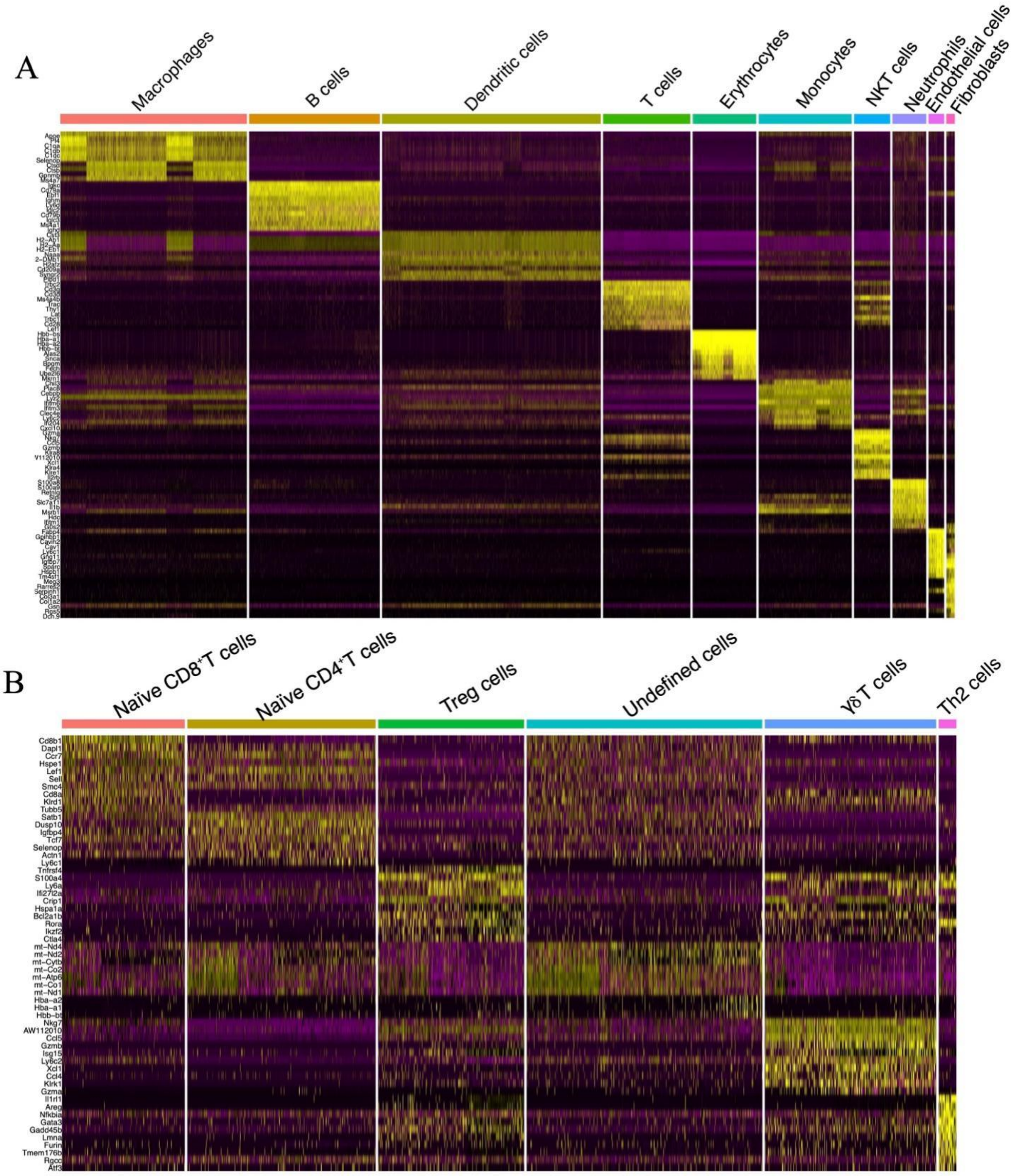
A
Macrophages
B cells
Dendritic cells
T cells
Erythrocytes
Monocytes
NKT cells
Neutrophils
Endothelial cells
Fibroblasts
B
Naïve CD8⁺T cells
Naïve CD4⁺T cells
Treg cells
Undefined cells
γδ T cells
Th2 cells
Cd8b1
Dapl1
Ccr7
Hspe1
Lef1
Sell
Smc4
Cd8a
Klrd1
Tubb5
Satb1
Dusp10
Igfbp4
Tcf7
Selenop
Actn1
Ly6c1
Tnfrsf4
S100a4
Ly6a
Ifi27l2a
Crip1
Hspa1a
Bcl2a1b
Rora
Ikzf2
Ctla4
mt-Nd4
mt-Nd2
mt-Cytb
mt-Co2
mt-Atp6
mt-Co1
mt-Nd1
Hba-a2
Hba-a1
Hbb-bt
Nkg7
AW112010
Ccl5
Gzmb
Isg15
Ly6c2
Xcl1
Ccl4
Klrk1
Gzma
Il1rl1
Areg
Nfkbia
Gata3
Gadd45b
Lmna
Furin
Tmem176b
Rgcc
Atf3

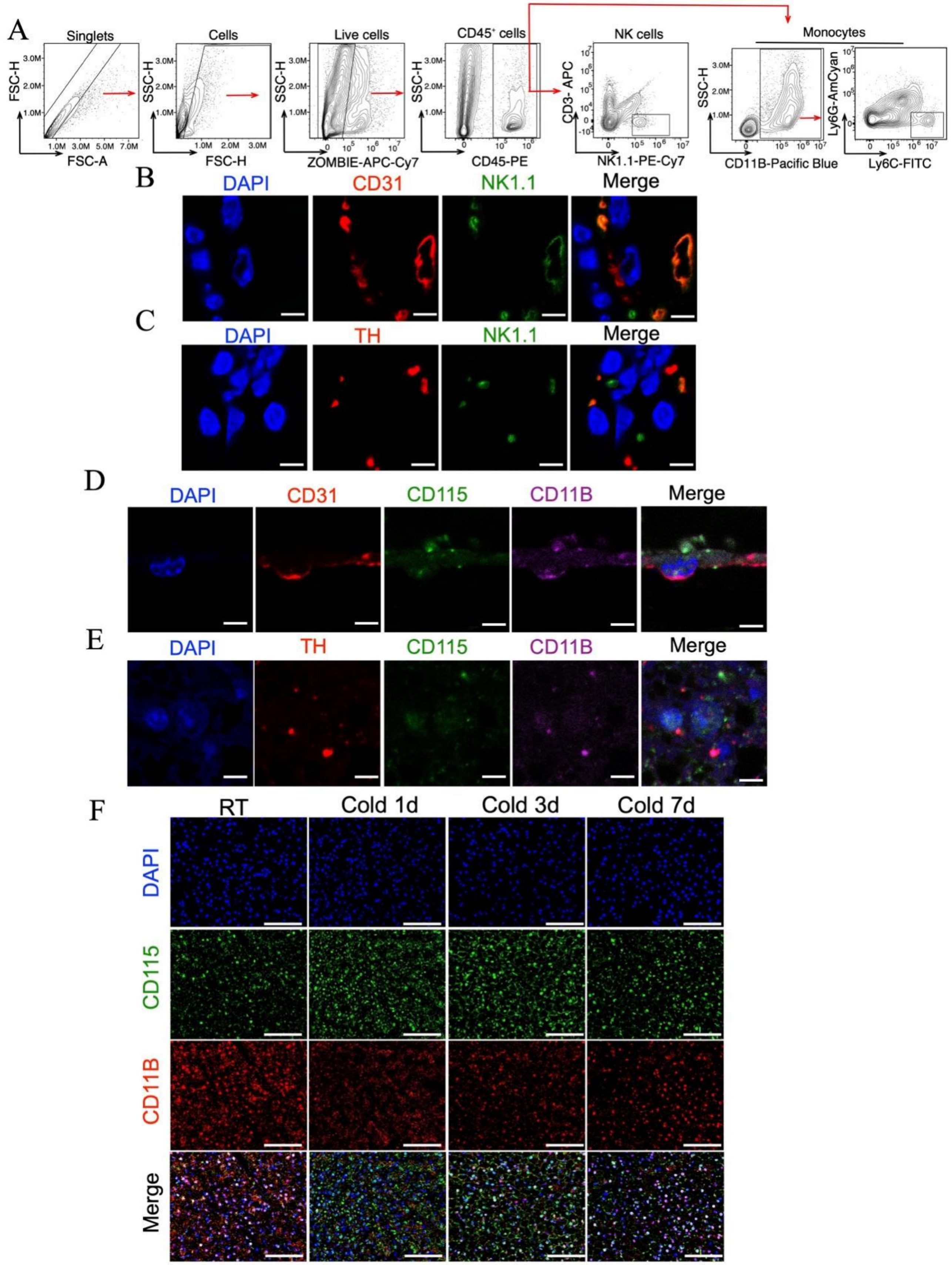
A
Singlets
FSC-H
FSC-A
Cells
SSC-H
FSC-H
Live cells
ZOMBIE-APC-Cy7
CD45+ cells
CD45-PE
NK cells
CD3- APC
NK1.1-PE-Cy7
Monocytes
CD11B-Pacific Blue
Ly6G-AmCyan
Ly6C-FITC
B
DAPI
CD31
NK1.1
Merge
C
DAPI
TH
NK1.1
Merge
D
DAPI
CD31
CD115
CD11B
Merge
E
DAPI
TH
CD115
CD11B
Merge
F
RT
Cold 1d
Cold 3d
Cold 7d
DAPI
CD115
CD11B
Merge

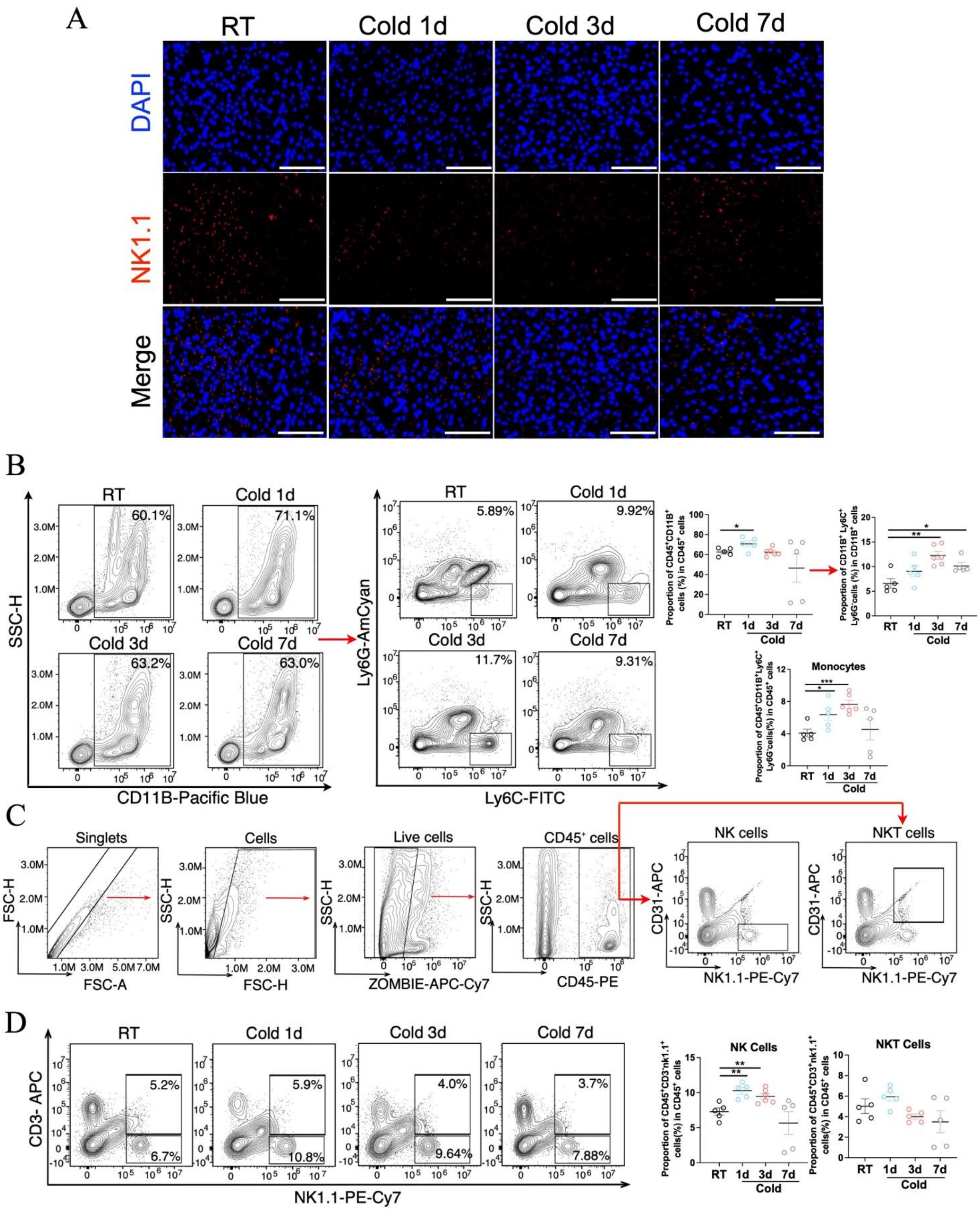
A
RT
Cold 1d
Cold 3d
Cold 7d
DAPI
NK1.1
Merge
B
RT
Cold 1d
Cold 3d
Cold 7d
60.1%
71.1%
63.2%
63.0%
SSC-H
CD11B-Pacific Blue
5.89%
9.92%
11.7%
9.31%
Ly6G-AmCyan
Ly6C-FITC
Proportion of CD45+CD11B+ cells (%) in CD45+ cells
Proportion of CD11B+ Ly6C+ Ly6G-cells (%) in CD11B+ cells
Monocytes
Proportion of CD45+CD11B+Ly6C+ Ly6G-cells(%) in CD45+ cells
RT
1d
3d
7d
Cold
C
Singlets
Cells
Live cells
CD45+ cells
NK cells
NKT cells
FSC-H
FSC-A
SSC-H
ZOMBIE-APC-Cy7
CD45-PE
CD31-APC
NK1.1-PE-Cy7
D
RT
Cold 1d
Cold 3d
Cold 7d
5.2%
6.7%
5.9%
10.8%
4.0%
9.64%
3.7%
7.88%
CD3- APC
NK1.1-PE-Cy7
NK Cells
NKT Cells
Proportion of CD45+CD3-nk1.1+ cells(%) in CD45+ cells
Proportion of CD45+CD3+nk1.1+ cells(%) in CD45+ cells

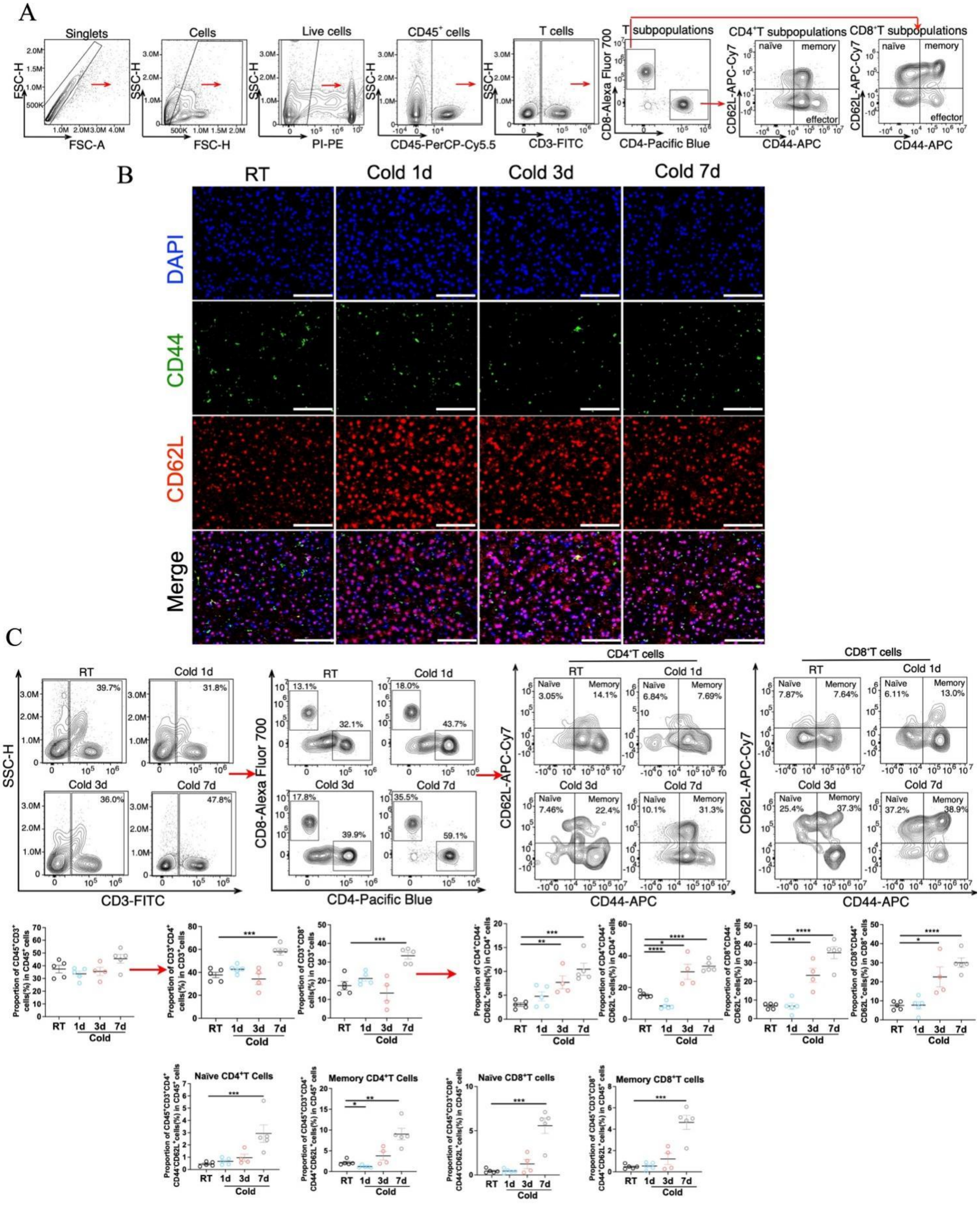
A
Singlets
Cells
Live cells
CD45+ cells
T cells
T subpopulations
CD4+T subpopulations
CD8+T subpopulations
FSC-H
FSC-A
SSC-H
PI-PE
CD45-PerCP-Cy5.5
CD3-FITC
CD8-Alexa Fluor 700
CD4-Pacific Blue
CD62L-APC-Cy7
CD44-APC
naïve
memory
effector
B
RT
Cold 1d
Cold 3d
Cold 7d
DAPI
CD44
CD62L
Merge
C
RT
Cold 1d
Cold 3d
Cold 7d
39.7%
31.8%
36.0%
47.8%
13.1%
32.1%
18.0%
43.7%
17.8%
39.9%
35.5%
59.1%
CD4+T cells
CD8+T cells
Naïve
Memory
3.05%
14.1%
6.84%
7.69%
7.46%
22.4%
10.1%
31.3%
7.87%
7.64%
6.11%
13.0%
25.4%
37.3%
37.2%
38.9%
SSC-H
CD3-FITC
CD8-Alexa Fluor 700
CD4-Pacific Blue
CD62L-APC-Cy7
CD44-APC
Proportion of CD45+CD3+ cells(%) in CD45+ cells
Proportion of CD3+CD4+ cells(%) in CD3+cells
Proportion of CD3+CD8+ cells(%) in CD3+cells
Proportion of CD4+CD44- CD62L+cells(%) in CD4+ cells
Proportion of CD4+CD44+ CD62L+cells(%) in CD4+ cells
Proportion of CD8+CD44- CD62L+cells(%) in CD8+ cells
Proportion of CD8+CD44+ CD62L+cells(%) in CD8+ cells
Naïve CD4+T Cells
Memory CD4+T Cells
Naïve CD8+T cells
Memory CD8+T cells
RT
1d
3d
7d
Cold

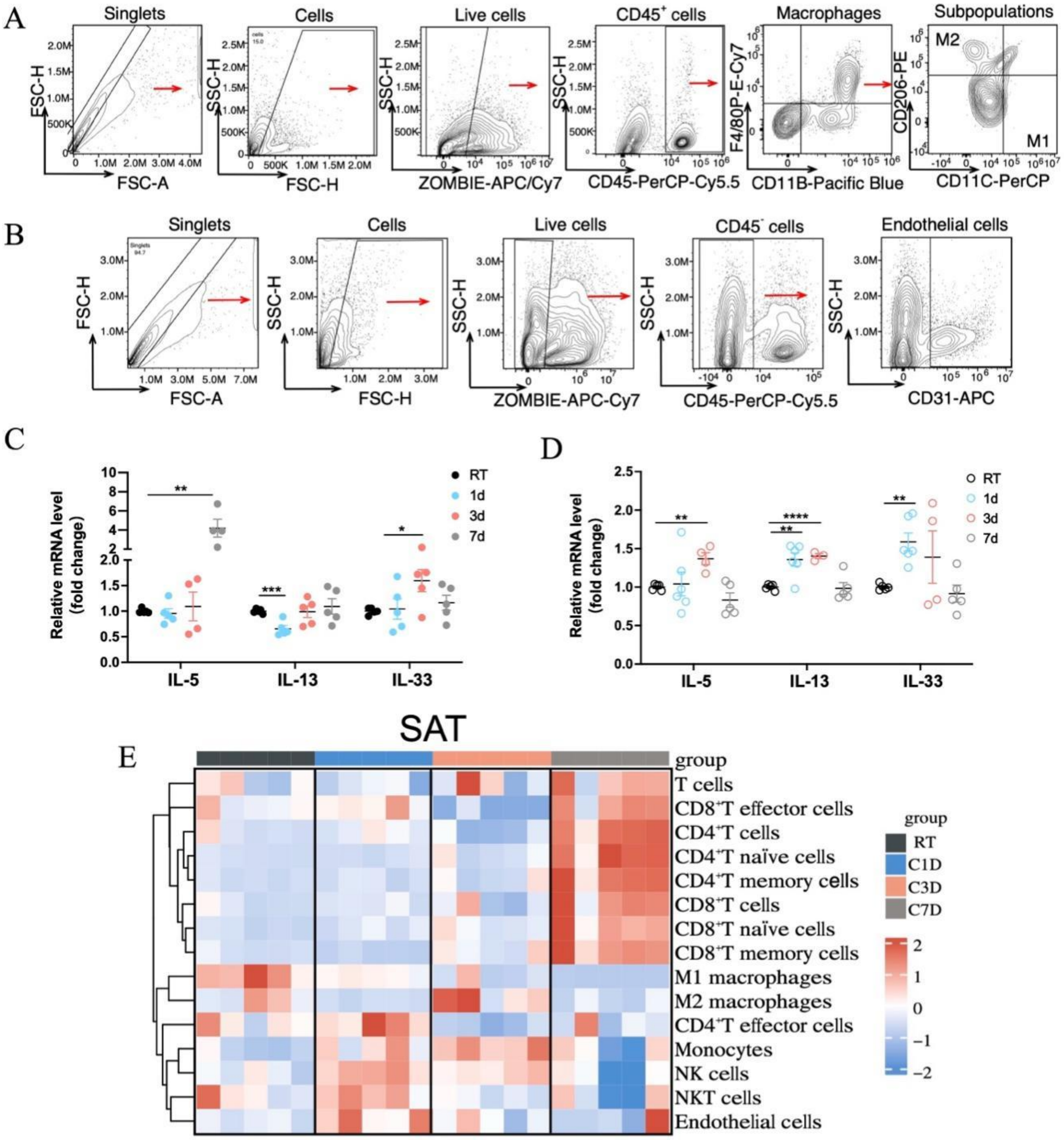
A
Singlets
Cells
Live cells
CD45⁺ cells
Macrophages
Subpopulations
FSC-H
FSC-A
SSC-H
FSC-H
ZOMBIE-APC/Cy7
CD45-PerCP-Cy5.5
F4/80P-E-Cy7
CD11B-Pacific Blue
CD206-PE
CD11C-PerCP
M2
M1
B
Singlets
Cells
Live cells
CD45⁻ cells
Endothelial cells
FSC-H
FSC-A
SSC-H
FSC-H
ZOMBIE-APC-Cy7
CD45-PerCP-Cy5.5
CD31-APC
C
D
Relative mRNA level (fold change)
RT
1d
3d
7d
IL-5
IL-13
IL-33
E
SAT
group
T cells
CD8⁺T effector cells
CD4⁺T cells
CD4⁺T naïve cells
CD4⁺T memory cells
CD8⁺T cells
CD8⁺T naïve cells
CD8⁺T memory cells
M1 macrophages
M2 macrophages
CD4⁺T effector cells
Monocytes
NK cells
NKT cells
Endothelial cells
RT
C1D
C3D
C7D
